# Light Chain Isotype and Antibody-Specificity Impact on Virus Neutralization

**DOI:** 10.3390/antib14020050

**Published:** 2025-06-17

**Authors:** Lin Sun, Roman Palt, Georg Schütz, Esther Föderl-Höbenreich, Laura Brod, Antonia Hermle, Anja Lux, Herta Steinkellner, Somanath Kallolimath

**Affiliations:** 1 Department of Biotechnology and Food Sciences, Institute of Plant Biotechnology and Cell Biology, BOKU University, Muthgasse 18, 1190 Vienna, Austria; 2 Core Facility Biomolecular & Cellular Analysis, BOKU University, 1190 Vienna, Austria; 3 Diagnostic and Research Institute of Pathology, Medical University of Graz, 8010 Graz, Austria; 4 Division of Genetics, Department of Biology, Friedrich-Alexander-Universität Erlangen-Nürnberg, Erwin-Rommel-Str. 3, 91058 Erlangen, Germany; 5 FAU Profile Centre Immunomedicine, Erwin-Rommel-Str. 3, 91058 Erlangen, Germany

**Keywords:** kappa–lambda light chain, monoclonal antibodies, SARS-CoV-2, Fcγ-receptor binding, virus neutralization, stability

## Abstract

Therapeutic antibodies with lambda light chains (λ-Abs) are underrepresented compared to kappa light chains (κ-Abs). Here, we evaluated two SARS-CoV-2-specific monoclonal antibodies (mAbs) that exhibit high (P5C3) and low (H4) antigen binding as κ and λ variants. mAbs expressed in glycoengineered *Nicotiana benthamiana* did not show differences in expression levels, glycosylation, and antigen binding, while κ-Abs exhibited slightly increased thermodynamic stability over λ-Abs. SARS-CoV-2 neutralization and IgG-FcγR immune complex studies revealed increased activities of H4 IgG1κ compared to H4 IgG1λ, with no differences observed between P5C3 variants. Our results indicate that constant light chain variability and Ab specificity contribute to Ab features, a fact that should be considered in engineering therapeutics.

## 1. Introduction

Antibodies (Abs) are becoming increasingly valuable tools in research and biomedical applications due to their unique combination of diversity, high specificity, stability, and compatibility with reliable production systems. Strategic engineering is being intensively applied to (i) gain a better understanding of Ab-associated activities [1,2] and, based on that knowledge, to (ii) generate novel formats with innovative functionalities [3]. Numerous studies were performed to elucidate functional activities of the heavy chain (HC) domains down to the single amino acid level with amazing success (reviewed by [4,5,6]). Less is known about the impact of light chains (LCs), the second polypeptide required for the assembly of intact Abs. In contrast to the HC, which appears in multiple sub-, iso-, and allotypes, the LC exists only in two isoforms, i.e., lambda (λ) and kappa (κ). In humans, lambda light chains contribute to antibody diversity through subtypes encoded by four IGLCs (IGLC1-3 and 7), whereas IGLC4-6 are pseudogenes. The kappa light chain has only one subtype and exhibits diversity through three known alleles: Km1, Km1,2, and Km3. These allotypic variants are encoded by specific IGKC genes: Km1 corresponds to IGKC6, Km1,2 to IGKC4, and Km3 to four IGKC alleles—IGKC1-3 and 5. The Km3 kappa light chain allotype was chosen for antibody generation due to its high prevalence and immunogenetic relevance in the human population. Its widespread expression minimizes the risk of allotype-specific immunogenicity. Km3 provides a stable and well-characterized scaffold that supports efficient expression and folding in recombinant systems. Its use aligns with established antibody platforms, facilitating consistency and comparability across studies. These attributes make Km3 a suitable and reliable choice for therapeutic antibody development and analysis. Unlike kappa (κ) light chains, lambda (λ) light chains have not been similarly characterized for allotypic variation [7]. The two LC isotypes are unevenly distributed in mammals and occur in human immunoglobulins in a ratio of ~2:1 in favor of κ [8]. Although it is currently unclear whether the use of one LC offers an advantage over another, 90% of biotechnologically produced mAbs are κ-Abs [9]. Reasons for this λ paucity are unclear but may be associated with unintended selection bias in screening library design [10] or the higher κ:λ ratios in humans and some other mammals [11]. Studies also show that λ-variable LCs (λ-VLs) exhibit higher hydrophobicity than κ-VLs, indicating an increased tendency for aggregate formation due to the hydrophobic effect [12,13]. However, the identification of an increasing number of epitopes that are preferentially activated by λ-Abs suggests that there are functional disadvantages if these epitopes are not considered in drug development. For example, it has been observed that κ- and λ-Abs are expressed in characteristic proportions with restricted usages, and that they tend to have different antigen specificities [14]. In agreement, a λ-LC bias for viral envelope protein epitopes associated with enhanced Ab function has been reported [15]. These improvements are attributed to longer CDRL3 in λ, but no systematic studies are available. Interestingly, despite the thousands of SARS-CoV-2 Abs explored during the COVID-19 pandemic, to the best of the authors’ knowledge, only one study addressed λ- and κ-LCs and found equal distribution of κ to λ within total virus-specific IgG, with a trend towards λ-Abs against nucleocapsid and spike proteins [16]. To gain functional insights, here, we aimed at a comparative investigation of two well-characterized SARS-CoV-2 monoclonal antibodies (mAb) produced as κ and λ variants.

## 2. Materials and Methods

### 2.1. Generation of IgG1 Expression Vectors

P5C3 and H4, two broadly neutralizing antibodies against SARS-CoV-2, were used in the present study. P5C3 can neutralize variants of concern, including omicron [17]. H4 can bind to the omicron BA.1 variant but does not neutralize [18]. The IgG1 heavy and corresponding kappa light chain (HC and ĸLC) constructs were generated as described previously [19,20]. To generate a lambda light chain (λLC), codon-optimized variable light chain fragments of *P5C3-LCFv* (324 bp) or *H4-LCFv* (339 bp) were inserted into the MagnICON^®^ vectors carrying barley alfa amylase signal peptide and light chain constant domains of IgG lambda isotypes (λLc, 318 bp), resulting in full-length constructs of *P5C3/H4-IgG-*λ*LC* (642 bp/657 bp). The resulting vectors were transferred to *Agrobacterium tumefaciens* (strain GV3101 pMP90) and used for subsequent agroinfiltration experiments. The primer pairs used to generate constructs are listed in Supplementary Table S1, and the schematic representation of IgG1ĸ and IgG1λ mAbs, P5C3 and H4 IgG1 peptide sequences are shown in Supplementary Figures S1 and S2.

### 2.2. Expression and Purification of IgG1 Antibody Isotypes

For the production of P5C3/H4-IgG1, glycoengineered *Nicotiana benthamiana* (ΔXTFT), lacking plant-specific core xylose and fucose residues, was used [21]. The plants were grown in a growth chamber at 24 °C and 60% humidity, with a 16 h light/8 h dark photoperiod. To produce IgG1 isotypes, liquid cultures of agrobacteria transformed with *magnICON* vectors [22] carrying genes for P5C3/H4-IgG1HC, P5C3/H4-IgGκLC, and P5C3/H4-IgGλLC were grown at 29 °C for 24 h, centrifuged at 4000× *g* for 10 min, and resuspended in an infiltration buffer (10 mM MgSO_4_; 10 mM MES; pH 5.6). The final optical density (OD_600_) of each construct was set to 0.1. The agroinfiltration mix is delivered to the leaves of 4–5-week-old plants by syringe infiltration. Four days post-infiltration (dpi), leaves were harvested, immediately flash-frozen in liquid nitrogen, and ground into a fine powder. Total soluble proteins (TSPs) were extracted using an extraction buffer composed of 0.5 M NaCl, 45 mM Tris, 1 mM EDTA, and 40 mM ascorbic acid (pH 7.4), at a ratio of 1:2 (*w*/*v*; fresh leaf weight to buffer). The mixture was incubated for 90 min at 4 °C with continuous shaking on an orbital shaker. After extraction, the homogenate was centrifuged twice at 14,000× *g* for 20 min at 4 °C to remove debris. The supernatant was then vacuum-filtered sequentially through 8–12 µm and 2–3 µm filter papers (ROTILABO^®^ Typ 12A and 15A).

Recombinant P5C3/H4 IgG1 antibodies were purified from the TSP using protein A affinity chromatography (rProA, Amicogen Inc., Gimhae, Gyeongsangnam-do, Republic of Korea; Cat. No. 1080025). The clarified extract was loaded onto a manually packed column pre-equilibrated with 10 column volumes of 1× PBS (137 mM NaCl, 3 mM KCl, 10 mM Na_2_HPO_4_, and 1.8 mM KH_2_PO_4_; pH 7.4) at a flow rate of 1.5 mL/min. Washing was carried out with 20 CV of 1× PBS (pH 7.4). Antibodies were eluted in 1 mL fractions with 0.1 M Glycine/HCl (pH 2.5), and eluates were immediately neutralized with 1 M Tris (pH 9.0) and dialyzed overnight against 1× PBS (pH 7.4). IgG1 isotypes were separated by size-exclusion chromatography using a HiLoad Sephadex 200/10/300 GL column (GE Healthcare Bio-Sciences AB, Uppsala, Sweden). The column was pre-equilibrated and eluted with 1.5 column volumes of elution buffer (1× PBS containing 200 mM NaCl, pH 7.4) at a flow rate of 0.4 mL/min. Fractions corresponding to monomeric full-length IgG1 were collected and subsequently concentrated using Amicon Ultra centrifugal filters with a 10 kDa molecular weight cutoff (Merck Millipore, Darmstadt, Germany, UFC5010). Protein concentrations were measured using a NanoDrop™ 2000 spectrophotometer (Thermo Scientific, Wilmington, NC, USA). SDS-PAGE analyses were performed in a 12% or 4–20% gradient acrylamide gel under reducing or non-reducing conditions. Gels were stained with Coomassie Brilliant Blue R 250 (Carl Roth GmbH Co. KG, Karlsruhe, Germany).

### 2.3. N-Glycan Analysis

The N-glycosylation profiles of the purified Abs were determined by mass spectrometry (MS) as described previously [23,24]. Briefly, approximately 6 µg of purified antibodies was trypsin-digested and analyzed with an LC-ESI-MS system (Thermo Orbitrap Exploris 480, Pleasanton, CA, USA). The possible glycopeptides were identified as sets of peaks consisting of the peptide moiety and the N-glycan varying in the number of HexNAc units, as well as hexose, deoxyhexose, and pentose residues attached to a peptide. FreeStyle 1.8 (Thermo, Pleasanton, CA, USA) was used to search glycopeptides, and deconvolution was performed by the extract function. The peak heights roughly reflect the molar ratios of the glycoforms. Nomenclature according to the Consortium for Functional Glycomics [25] was used.

### 2.4. Thermal Stability of IgG1 Antibody Isotypes

Differential scanning calorimetry (DSC) was performed using a MicroCal PEAQ-DSC automated device (Malvern Panalytic Ltd., Worcestershire, UK). The concentration of all four IgG1 isotypes stored in a PBS buffer at pH 7.4 was adjusted to 1 mg/mL (6.7 µM). For the protein scans, 325 µL of each sample was heated from 20 °C to 100 °C at a scan rate of 60 °C/h under ~4.1 bar cell pressure using the high-feedback mode. Before each scan, a pre-scan thermostat was set at 20 °C for 5 min. After the first scan, each sample was cooled and reheated again under the same settings. The re-scans were used for buffer subtraction, and the baselines were then further corrected with the progress baseline parameters. Analysis and fitting of the transition estimates using the Non-Two-State model was carried out on MicroCal PEAQ-DSC software (Version 1.61) supplied by the manufacturer.

### 2.5. Direct Sandwich ELISA

Direct sandwich ELISAs were carried out in detail as previously described [19]. Using SARS-CoV-2 spike protein RBD_215_ descending from the Wuhan variant [26] as the antigen, and HRP-conjugated mAb CR3022 as a secondary antibody (1:15,000 blocking solution; 3% fat-free milk in PBST), analyses were performed. Purified mAbs were diluted in 1× PBS (pH 7.4) to 0.5 µg/mL and 2 µg/mL of P5C3- and H4-IgG1, respectively, with 50 µL/well in 96-well microplates (Thermo Fischer Maxisorp, Roskilde, Denmark, catalog No.: M9410-1CS). RBD was diluted in a blocking solution and applied to the mAb-coated plate in a two-fold serial dilution starting from 500 ng/mL (P5C3) and 4000 ng/mL (H4). The intermediate washing steps were performed using 0.05% Tween in PBST. For detection, 50 µL/well of 3,3′,5,5′-tetramethylbenzidine (Thermo Fisher, Schnelldorf, Germany J61325. AU) was used. The reaction was stopped after 5 min with 50 µL/well of 2 M H_2_SO_4_. Absorbance was recorded at 450 nm with a reference wavelength of 620 nm using a Tecan Spark^®^ spectrophotometer, (Tecan Trading AG, Männedorf, Switzerland). Each sample was analyzed with a minimum of two technical replicates. EC_50_ values were determined by nonlinear regression of blank-corrected data using a four-parameter logistic (4PL) model in GraphPad Prism (version 9).

### 2.6. SARS-CoV-2 Neutralization Assay

Neutralization assays were conducted as described [27]. Briefly, VeroE6 cells (VC-FTV6, Biomedica, Vienna, Austria) were seeded in 48-well plates to reach 100% confluency by the time of infection. The P5C3- or H4-IgG1 light chain isotype mAbs were serially diluted (2-fold) and incubated with SARS-CoV-2 (Delta variant GK/478K.V1 (B.1.617.2 + AY.x); GISAID name: hCoV-19/Austria/Graz-MUG21/2021) for 30 min at 37 °C. Two wells were infected with the same mAb/SARS-CoV-2 mixture, while two additional wells were treated with SARS-CoV-2 alone, serving as a positive control. The cells were incubated at 37 °C for 1 h. Following incubation, the inoculum was removed, and the cells were overlaid with 1.5% carboxymethyl cellulose (Sigma-Aldrich, St. Louis, MO, USA). Then, 48 h post-incubation, cells were fixed with 4% neutral buffered formalin and immunohistochemically stained as previously described [23]. Plaque counts in the positive control were set as 100%. The inhibitory concentration (IC_50_) was calculated using normalized data for nonlinear regression analysis with variable slopes (GraphPad PRISM Version 9). All experimental procedures involving SARS-CoV-2 were performed in a BSL-3 laboratory.

### 2.7. Flow Cytometric Immune Complex Binding Assay

P5C3 and H4 binding to human primary leukocytes was assessed as previously described [28]. Briefly, 2 × 10^9^ streptavidin-coated fluorescent microspheres (Dragon Green, 200 nm, Bangs Laboratories, Fishers, IN, USA) were mixed with 1.5 µg of biotin-labeled SARS-CoV-2 RBD derived from the Wuhan Hu-1 strain (recombinant SARS-CoV-2 spike protein receptor-binding domain encompassing amino acids 319–541, Biomol, Hamburg, Germany) following blocking in a blocking solution (Invitrogen, Eugene, OR, USA). Opsonized microspheres were incubated for two hours at 50 rpm RT, followed by storage at 4 °C overnight. Excess RBD antigens were washed off by centrifugation, followed by the addition of 5.0 µg/mL of P5C3 or 20.0 µg/mL of H4 antibody variants (to account for differential binding in RBD’s binding affinity). Microspheres were washed with PBS after incubation for 2 h at 50 rpm before resuspension in PBS. Subsequently, opsonized microspheres were incubated with 5 × 10^5^ peripheral blood leukocytes (PBLs) obtained by erythrocyte lysis from the peripheral blood of healthy donors (under approval from the Ethics Committee of FAU Erlangen and after volunteers providing written informed consent to participate in this study). Microspheres only coated with the antigen (RBD) and PBS were used as negative controls. Cells were incubated for 2 h on ice and gently shaken before staining of surface markers to detect individual leukocyte populations. In the absence of staining for CD16, an additional marker to distinguish NK cells and monocyte subsets whose staining would have interfered with the binding assay itself, populations were identified as follows: CD14^+^ classical monocytes, CD14^−^CD33^+^ non-classical monocytes, CD19^+^ B cells, CD56^+^CD3^−^ NK cells, and CD33^+^SSC^high^ neutrophils within the living (DAPI^−^) CD45^+^ cell population upon the exclusion of duplets. All antibodies were acquired from Biolegend, San Diego, CA, USA: Brilliant Violet 510 anti-human CD14 (clone: M5E2), APC anti-human CD33 (clone: WM53), PE/Cy7 anti-human CD19 (clone: SJ25C1), PE anti-human CD3 (clone: UCHT), PE-Cyanine5 anti-human CD56 (clone: MEM-188), and APC/Fire™ 750 anti-human CD45 (clone: 2D1). Data was acquired on BD FACSCanto II and analyzed with FlowJo software version 10.8.1. The percentage of bead-positive cells was calculated within each subpopulation. Experiments were performed with PBL from six different donors.

## 3. Results

### 3.1. Expression, Purification, and Biophysical Characterization of Antibody Isotypes

**Figure 1:**
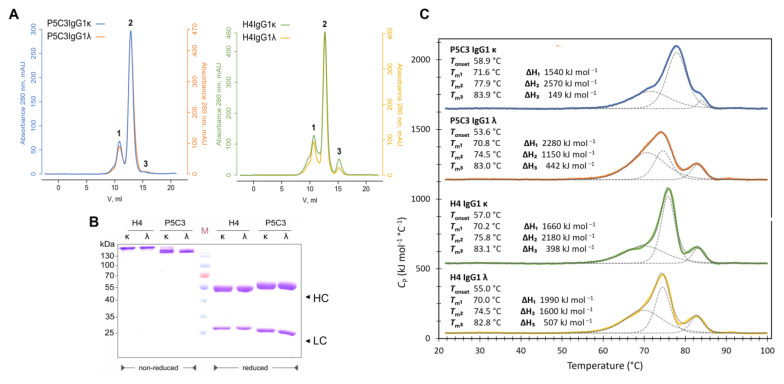
Biophysical characterization of H4- and P5C3 IgG1. (**A**) SEC profiles of protein A-purified mAbs. According to the elution order, the peaks were assigned as follows: (1) aggregates, (2) fully assembled IgG1 monomers, and (3) degradation products. The *Y*-axis represents light absorbance units (mAU) at 280 nm; the *X*-axis represents elution volume in mL. (For a simplified comparison, mAb profiles are scaled individually). (**B**) SDS-PAGE of purified H4- and P5C3-IgG1 ĸ and λ Abs, respectively (left: non-reducing conditions; right: reducing conditions). LC and HC at approximately 25 and 55 kDa, respectively. Fully assembled mAbs at position ~ 180 kDa. (**C**) The thermodynamic stability of mAbs was measured by DSC. Thermograms of P5C3 IgG1κ (blue), P5C3 IgG1λ (orange), H4 IgG1κ (green), and H4 IgG1λ (yellow) with the corresponding fits of the endotherms (dashed and solid lines in gray). The values of the fitting parameters (T_onset_, Tm, and ∆H) are shown above each thermogram. Conditions: 6.7 µM IgG1s, 1× PBS, pH 7.4, and heating rate 60 °C h^−1^. The thermograms were shifted for better visibility by 1640, 1140, 540, and 40 kJ mol^−1^ °C^−1^.

Anti-SARS-CoV-2 mAbs P5C3 and H4 served as templates in this study. Both mAbs are derived from convalescent human sera and were originally expressed as IgG1 and κ LC isotypes (IgG1κ; [29,30]). The antibodies differ significantly in their ability to bind to nonoverlapping epitopes in the receptor-binding domain (RBD) of the spike protein. While P5C3 exhibits binding affinities in the picomolar range, these values are orders of magnitude higher for H4, depending on the virus isolate. This also translates to virus neutralization activities [29,30] even when expressed in IgG3 and IgM formats [19,24]. Here, a potent transient expression approach was used for the comparative expression of the two antibodies in the κ and λ formats. The corresponding P5C3-, H4-IgG1-HC-, and LC- gene constructs were transiently co-expressed in the glycosylation mutant *Nicotiana benthamiana* line ΔXTFT, lacking plant-specific core xylose and fucose [21]. mAbs were purified with protein A affinity chromatography followed by size-exclusion chromatography (SEC), resulting in four mAb formats: P5C3IgG1ĸ, P5C3IgG1λ, H4IgG1ĸ, and H4IgG1λ (Figures S1 and S2). Purification yields of all four Ab variants, which were comparable, ranging between 220 and 300 µg mAb/g of leaf material (Table S2), and SDS-PAGE confirmed the presence of the LC and HC without obvious degradation products. All four mAbs efficiently assembled into heterodimers (Figure 1A,B). The N-glycosylation status of mAbs was determined by liquid chromatography–electrospray ionization–tandem mass spectrometry (LC-ESI-MS/MS). MS spectra for all four mAbs displayed a single dominant glycoform at the Fc glycosylation site, namely xylose and core fucose-free GlcNAc-terminated structures (between 90 and 92% GnGn/GnM structures), accompanied by ~8% mannosidic structures. Such profiles are typical for ΔXTFT-produced IgGs [21] (Figure S3). Collectively, biochemical analyses did not exhibit obvious differences associated with ĸ or λ chains.

The thermodynamic stability and the unfolding mechanism of the four mAbs were investigated using differential scanning calorimetry (DSC). The thermograms of temperature-induced mAb unfolding are shown in Figure 1C. All four profiles consist of three transitions as either independent peaks or distinct shoulders. These typical three unfolding transitions are assigned to CH2, Fab, and CH3 domains in ascending temperature [31,32,33]. Here, the differences in κ and λ-Abs lie solely in the constant LC (CLC) domain; hence, major variations in the transition are expected in the Fab. The T_m_ of the Fab transitions lies at 3.4 °C (Tm2s 74.5–77.9 °C) for all four IgG1s (Figure 1C), whereas the melting temperature variation in CH2 and CH3 domains in all four mAbs is only within 1.6 °C (Tm1s 70.0–71.6 °C) and 1.1 °C (Tm3s 82.8–83.9 °C), respectively.

A comparison between κ-mAbs showed that P5C3 is more stable than H4 with higher melting temperatures across all three transitions, while λ-mAbs did not show an obvious difference. When comparing the κ and λ isotypes, we observed that κ contributes to a higher stability regarding the Tm of all three transitions in both H4 and P5C3. The stabilizing effect of κ over λ is also noticeable in a higher T_onset_ for both mAbs.

### 3.2. Antigen-Binding and Virus-Neutralization Activity of Antibody Isotypes

**Figure 2:**
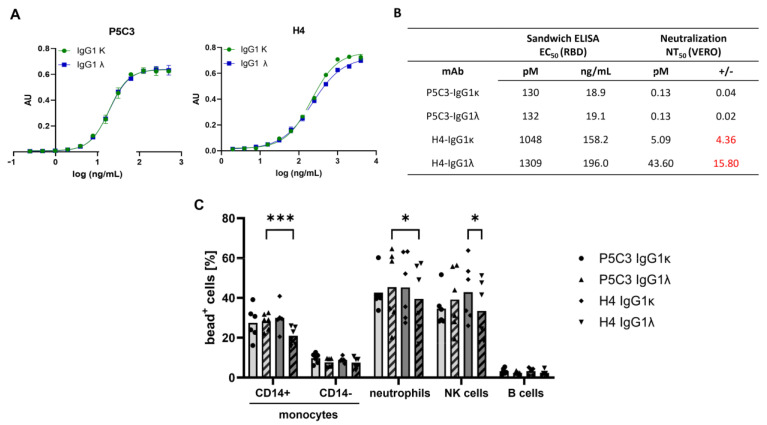
Functional characterization of P5C3-H4 and H4- IgG1. (**A**) Sandwich ELISA: binding activity of purified mAbs to recombinant RBD, using CR3022-HRP for detection. *X*-axis: concentration (ng/mL); *Y*-axis: absorbance (AU). (**B**) Ag-binding EC_50_ and neutralization NT_50_ values. Independent duplicates. The deviations observed in neutralization assay is highlighted in red. (**C**) Flow cytometric binding assay of IgG immune complexes to primary human leukocytes: Following incubation of microspheres with peripheral blood-derived human leukocytes, flow cytometry was performed, and the proportion of microsphere-positive cells was calculated within the individual populations (% bead^+^ cells on the *Y*-axis). Graphs show individual values and means of two independent experiments using a total of six donors. Fluorescent microspheres were coated with RBD before the addition of RBD-specific P5C3 or H4 mAb variants. Statistical analysis was performed by a two-way ANOVA and Tukey’s multiple comparisons post hoc test. * *p* < 0.05 and *** *p* < 0.001.

Next, mAbs were investigated for their antigen-binding and neutralization abilities. Direct sandwich ELISAs using the SARS-CoV-2 spike protein RBD (Wuhan strain) [19] exhibited an 8- to 10-fold greater binding activity of P5C3 compared to H4 variants (EC_50_ of 130 pM and 1048 pM for ĸ isotypes). This feature also translated to the λ-Abs; no obvious alterations associated with the LC isotypes were observed. To determine the neutralization activities, Vero cell-based SARS-CoV-2 plaque reduction assays [27] were performed. No differences in NT potency were noticed by comparing high-antigen-binding P5C3 IgG1ĸ and λ, with IC_50_ ~0.13 pM (Figure 2B). In contrast, for the low-antigen-binding H4 IgG1, the ĸ isotype exhibited a ~8-fold increased NT activity compared to the λ isotype (IC_50_: H4 IgG1ĸ: 5 pM; H4 IgG1λ: 43 pM; Figure 2B).

### 3.3. IgG Immune Complex Binding to Primary Human Leukocytes

Finally, a highly sophisticated approach was used to investigate the interaction of the IgG1-Fc with specific Fcγ receptors (FcγR). The approach relies on the prior complexation of the mAbs with RBD, resulting in the formation of multivalent immune complexes (ICs), closely resembling IgG-opsonized viral particles [28,34]. In addition to NK and B cells that express either activating FcγRIIIa or inhibitory FcγRIIb, respectively, leukocyte subsets (CD14^+^ classical and CD14^−^ non-classical monocytes), as well as neutrophilic granulocytes that express multiple (activating and inactivating) FcγRs, were used [35]. Generally, mAb binding was more pronounced on classical monocytes (CD14^+^), primary neutrophils, and NK cells compared to non-classical monocytes (CD14^−^) and B cells, consistent with previous findings [28]. Pairwise comparison of κ and λ light chain variants showed similar binding for P5C3 mAbs, whereas for H4, the λ variant exhibited slightly but significantly reduced binding compared to its κ counterpart. Moreover, P5C3 IgG1λ showed enhanced binding to CD14^+^ monocytes and neutrophils relative to H4 IgG1λ (Figure 2C).

## 4. Discussion

Here, we systematically investigated the contribution of the λ versus ĸ light chain of two mAbs with different specificities. Although increased production was previously observed for λ variants of IgM and dimeric IgA2 [27], we did not detect differences upon LC switching in the production process. In accordance, no obvious differences were observed regarding mAb assembly and post-translational modification (i.e., glycosylation). Accordingly, antigen binding was not affected by the LC switch. DSC studies exhibited CLC-associated variations in Fab unfolding and an increased thermodynamic stability of κ-Abs. Increased stability of κ-Abs has been previously reported and was attributed to differences in the CDR L3 region of the variable light chain (VLC) [36]. However, as the λ- and κ-Abs used in this study carry identical VLCs, this attribution can be excluded. Therefore, these differences should be associated with the CLC.

The antigen binding was assessed using the RBD from the Wuhan strain, while neutralization assays were conducted with the Delta variant. Both ĸ and λ antibodies showed no noticeable differences in antigen binding or neutralization across these variants. However, the possibility of variant-specific effects cannot be ruled out when tested against a broader panel of viral strains. Interestingly, we observe a clear difference in the NT activity of λ and ĸ, but only for the low antigen binder H4. However, for completeness, it should be mentioned that the NT_50_ value of P5C3 is close to the limit of detection, and small NT differences may have been overlooked. It also should be noted that we used an FcγR-independent assay that determines NT without the influence of cellular receptors. Previous studies show that mAbs with identical variable domains, but different HCs and hinge domains, exhibit altered NTs [28,37]. It is hypothesized that hinge flexibility augments Ab-based neutralization potency by a crosslinking event, thus increasing the overall avidity of the spike–Ab interaction [38]. This phenomenon seems to be especially pronounced at viruses with low spike protein densities, as is the case for SARS-CoV-2 [39].

Increasing evidence points to the importance of the interplay of Ab NT and Fc-mediated activity in SARS-CoV-2 [40,41,42]. It is well known that subtle alterations in the HC can impact the structural integrity and conformation of the Ab, thereby changing the FcγR-binding abilities [4]. Systematic studies on the impact of LC isotypes on FcγR binding have not been performed so far. A recent simulation study suggests an interplay of LC isotypes with Fc conformation and hinge flexibility, which might influence binding to FcγRs [43]. Here, we determined immune cell binding to λ- and κ-Abs in the form of multivalent IC, mimicking an in vivo amplifying protective activity of neutralizing Ab through opsonization [44]. While no obvious CLC-associated differences were observed in the primary human leukocytes used, H4 IgG1κ showed increased binding to various cell types compared to H4 IgG1λ. Differential binding activities point to variations in FcγR-induced effector functions such as antibody-induced cellular cytotoxicity (ADCC) or phagocytosis (ADCP).

In summary, biochemical features seem to remain unchanged upon mAb CLC switching. Minor but significant differences were observed between λ and κ Abs in thermodynamic stability, IC-based cellular FcγR binding, and NT. Functional differences were especially pronounced for H4, which shows low antigen binding. We cannot currently explain the molecular mechanisms underlying these observations. However, the contribution of both variable and constant regions to the expression of specific antigen-binding region idiotypes has been known for several decades [45,46], and data on the impact of LCs on the Fc conformation and hinge flexibility are available [43]. Our results demonstrate that further research is needed to better understand how antibodies modulate functional activities, which in turn will contribute to the development of new innovative products.

## Data Availability

Additional supporting information may be found online in the Supplementary Materials Section at the end of the article.

## Supplementary Materials

The following supporting information can be downloaded at https://www.mdpi.com/article/10.3390/antib14020050/s1. Table S1. Primer list to generate heavy and light chain constant domains of antibody isotypes with flanking BsmBI (CGTCTC), facilitating introduction of variable fragment with BsaI (GGTCTC) restriction sites. Table S2. Yield of purified mAbs. Figure S1. Schematic representation of IgG1ĸ and IgG1λ. VL: variable light; VH: Variable heavy; CL: constant light; CHγ1-CHγ3: constant heavy gamma chain domain1-3; Fab: Antigen binding fragment; Fc: Crystalizable fragment. Blue dot: Conserved N-glycosite. Figure S2. Peptide sequences of IgG1ĸ and IgG1λ Black: variable heavy and light chain fragments, Blue: constant heavy chain, Red: constant kappa light chain, Purple: constant Lambda light chain, N-glycosite highlighted in bold and Red. Figure S3: A. LC-ESI-MS/MS–derived N-glycosylation profiles of purified P5C3-IgG1 ĸ and λ. Bars represent the relative abundance (%) of glycoforms present at the Fc GS. Blue: complex GlcNAc-terminating N-glycans (GnM-GnGn); green: mannosidic N-glycans (Man8-Man9). Orange: combines detected glycans below 3%. B. Occupied (blue) and non-occupied glycosite (grey) in %. Nomenclature according to Altmann et al. 2024 [25].
