# Supplementary material for: Light Chain Isotype and Antibody-Specificity Impact on Virus Neutralization
Source: Antibodies (Basel). 2025 Jun 17;14(2):50. doi: 10.3390/antib14020050 (PMC12189767; doi:10.3390/antib14020050)
Supplement: Supplementary file 1 [file antibodies-14-00050-s001.zip › antibodies-3666698-supplementary.pdf]

Light chain isotype and antibody-specificity impact on virus neutralization

Lin Sun[1], Roman Palt[1, †], Georg Schütz[2], Esther Föderl-Höbenreich[3], Laura Brod[1], Antonia Hermle[4], Anja Lux[4, 5], Herta Steinkellner[1] and Somanath Kallolimath[1*]

[1]Institute of Plant Biotechnology and Cell Biology, Department of Biotechnology and Food Sciences, BOKU University, Vienna, Austria

[2]Core Facility Biomolecular & Cellular Analysis, BOKU University, Vienna, Austria

[3]Diagnostic and Research Institute of Pathology, Medical University of Graz, Graz, Austria

[4]Division of Genetics, Department of Biology, Friedrich-Alexander-Universität Erlangen-Nürnberg, Erwin-Rommel-Str. 3, 91058 Erlangen, Germany.

[5]FAU Profile Centre Immunomedicine, Erwin-Rommel-Str. 3, 91058 Erlangen, Germany.

[†]Current address: Virusure Forschung und Entwicklung, Donaucitystraße 1, 1220 Vienna

***Correspondence:**

Somanath Kallolimath, somanath.kallolimath@boku.ac.at

Supplementary data

Table S1: Primer list to generate heavy and light chain constant domains of antibody isotypes with flanking BsmBI (CGTCTC), facilitating introduction of variable fragment with BsaI (GGTCTC) restriction sites

Primers	Sequence
IgG1(-Fv) -F1	GAG**CGTCTC**TAGGTA**GAGACC**GGGATCC**GGTCTC**TGCATCAACCAAAGGTCC
IgG1-R1	GCA**CGTCTC**AAAGCTCACTTTCCAGGAGAAAGAG
κLc(-Fv) -F1	GAG**CGTCTC**TAGGTA**GAGACC**GGATCC**GGTCTC**TCGTACTGTTGCAGCTC
κLc-R1	GCA**CGTCTC**AAAGCTTAGCATTCACCTCGATTAAAG
λLc(-Fv) -F1	GAG**CGTCTC**TAGGTA**GAGACC**GGGATCC**GGTCTC**TCAACCTAAGGCTGCTCC
λLc-R1	TATA**CGTCTC**TAAGCCTAGCTGCACTCAG

Table S2: Yield of purified mAbs

mAb	Yield (µg/g)
P5C3IgG1 κ	220
P5C3IgG1 λ	300
H4IgG1 κ	250
H4IgG1 λ	220

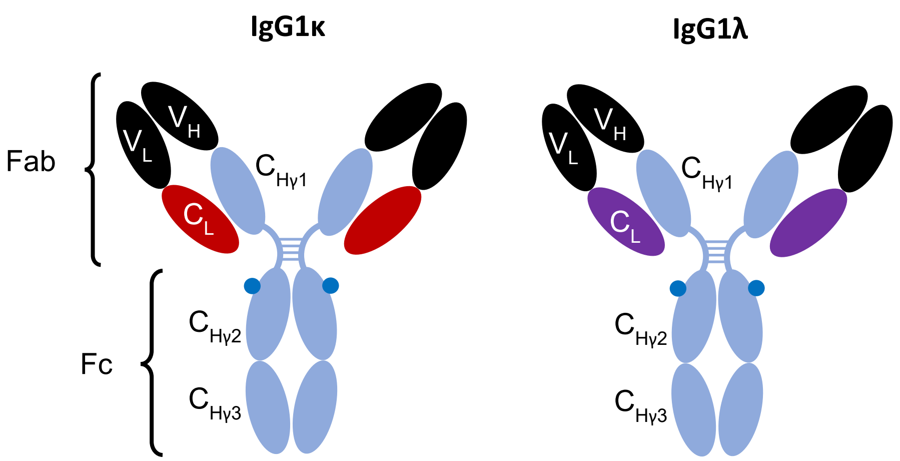

Figure S1: Schematic representation of IgG1κ and IgG1λ. VL: variable light; VH: Variable heavy; CL: constant light; CHγ1-CHγ3: constant heavy gamma chain domain1-3; Fab: Antigen binding fragment; Fc: Crystalizable fragment. Blue dot: Conserved N-glycosite

H4-IgG1Hc
QVQLVQSGAEVKKPGASVKVSCKASGYTFTGYYMHWVRQAPGQGLEWMGRINPNSGGTNYA
QKFQGRVTMTRDTSISTAYMELSRLRSDDTAVYYCARVPYCSSTSCHRDWYFDLWGRGTLVT
VSSASTKGPSVFPLAPSSKSTSGGTAALGCLVKDYFPEPVTVSWNSGALTSGVHTFPAVLQSS
GLYSLSSVVTVPSSSLGTQTYICNVNHKPSNTKVDKKAEPKSCDKTHTCPPCPAPELLGGPSVF
LFPPKPKDTLMISRTPEVTCVVVDVSHEDPEVKFNWYVDGVEVHNAKTKPREEQY**NST**YRVVS
VLTVLHQDWLNGKEYKCKVSNKALPAPIEKTISKAKGQPREPQVYTLPPSRDELTKNQVSLTCL
VKGFYPSDIAVEWESNGQPENNYKTTPPVLDSDGSFFLYSKLTVDKSRWQQGNVFSCSVMHE
ALHNHYTQKSLSLSPGK

H4-IgGκLc (allotype Km3)
DIQMTQSPLSLPVTPGEPASISCRSSQSLLDSDDGNTYLDWYLQKPGQSPQLLIYTLSYRASGV
PDRFSGSGSGTDFTLKISRVEAEDVGVYYCMQRIEFPLTFGGGTKVEIKRTVAAPSVFIFPPSDE
QLKSGTASVVCLLNNFYPREAKVQWKVDNALQSGNSQESVTEQDSKDSTYSLSSTLTLSKADY
EKHKVYACEVTHQGLSSPVTKSFNRGEC

H4-IgGλLc
DIQMTQSPLSLPVTPGEPASISCRSSQSLLDSDDGNTYLDWYLQKPGQSPQLLIYTLSYRASGV
PDRFSGSGSGTDFTLKISRVEAEDVGVYYCMQRIEFPLTFGGGTKVEIKQPKAAPSVTLFPPSS
EELQANKATLVCLISDFYPGAVTVAWKADSSPVKAGVETTTPSKQSNNKYAASSYLSLTPEQW
KSHRSYSCQVTHEGSTVEKTVAPTECS

P5C3-IgG1Hc
QMQLVQSGPEVKKPGTSVKVSCKASGFTFTSSAVQWVRQARGQRLEWIGWIVVGSGNTDYA
QQFQERVTITRDMSTSTAYMELSSLGSEDTAVYYCAAP**NCS**GGSCYDGFDLWGQGTMVTVSS
ASTKGPSVFPLAPSSKSTSGGTAALGCLVKDYFPEPVTVSWNSGALTSGVHTFPAVLQSSGLY
SLSSVVTVPSSSLGTQTYICNVNHKPSNTKVDKKAEPKSCDKTHTCPPCPAPELLGGPSVFLFP
PKPKDTLMISRTPEVTCVVVDVSHEDPEVKFNWYVDGVEVHNAKTKPREEQY**NST**YRVVSVLT
VLHQDWLNGKEYKCKVSNKALPAPIEKTISKAKGQPREPQVYTLPPSRDELTKNQVSLTCLVKG
FYPSDIAVEWESNGQPENNYKTTPPVLDSDGSFFLYSKLTVDKSRWQQGNVFSCSVMHEALH
NHYTQKSLSLSPGK

P5C3-IgGκLc (allotype Km3)
EIVLTQSPGTLSLSPGERATLSCRGSQSVRSSYLGWYQQKPGQAPRLLIYGASSRATGIPDRFS
GSGSGTDFTLTISRLEPEDFAVYYCQQYGSSPWTFGQGTKVEIKRTVAAPSVFIFPPSDEQLKS
GTASVVCLLNNFYPREAKVQWKVDNALQSGNSQESVTEQDSKDSTYSLSSTLTLSKADYEKHK
VYACEVTHQGLSSPVTKSFNRGEC

P5C3-IgGλLc
EIVLTQSPGTLSLSPGERATLSCRGSQSVRSSYLGWYQQKPGQAPRLLIYGASSRATGIPDRFS
GSGSGTDFTLTISRLEPEDFAVYYCQQYGSSPWTFGQGTKVEIKQPKAAPSVTLFPPSSEELQA
NKATLVCLISDFYPGAVTVAWKADSSPVKAGVETTTPSKQSNNKYAASSYLSLTPEQWKSHRS
YSCQVTHEGSTVEKTVAPTECS

Figure S2: Peptide sequences of IgG1κ and IgG1λ Black: variable heavy and light chain fragments, Blue: constant heavy chain, Red: constant kappa light chain, Purple: constant Lambda light chain, N-glycosite highlighted in bold and Red.

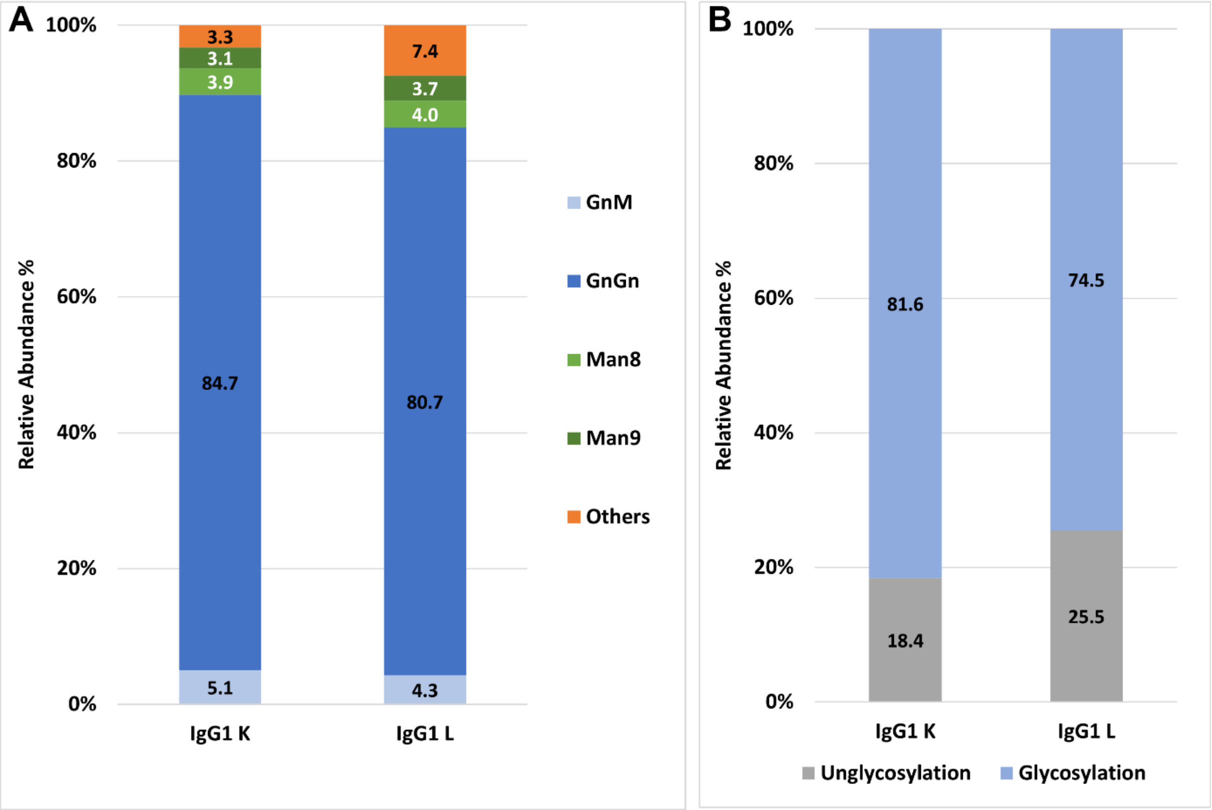

Figure S3: A. LC-ESI-MS/MS–derived N-glycosylation profiles of purified P5C3-IgG1 κ and λ. Bars represent the relative abundance (%) of glycoforms present at the Fc GS. Blue: complex GlcNAc-terminating N-glycans (GnM-GnGn); green: mannosidic N-glycans (Man8-Man9). Orange: combines detected glycans below 3%. **B.** Occupied (blue) and non-occupied glycosite (grey) in %. Nomenclature according to Altmann et al 2024.
